# Tissue distribution of cysteine string protein/DNAJC5 in *C. elegans* analysed by CRISPR/Cas9-mediated tagging of endogenous DNJ-14

**DOI:** 10.1007/s00441-024-03875-w

**Published:** 2024-02-26

**Authors:** Eleanor Barker, Alan Morgan, Jeff W. Barclay

**Affiliations:** 1 Department of Molecular Physiology & Cell Signalling, Institute of Systems, Molecular and Integrative Biology, University of Liverpool, Crown St, Liverpool, L69 3BX UK; 2 Current address: Division of Diabetes, Endocrinology and Gastroenterology, Faculty of Biology, Medicine and Health, The University of Manchester, Oxford Road, Manchester, M13 9PT UK

**Keywords:** CLN4, DNAJC5, DNJ-14, Kufs disease, Neurodegeneration, Parry disease

## Abstract

Cysteine string protein (CSP) is a member of the DnaJ/Hsp40 family of molecular chaperones. CSP is enriched in neurons, where it mainly localises to synaptic vesicles. Mutations in CSP-encoding genes in flies, worms, mice and humans result in neuronal dysfunction, neurodegeneration and reduced lifespan. Most attention has therefore focused on CSP’s neuronal functions, although CSP is also expressed in non-neuronal cells. Here, we used genome editing to fluorescently tag the *Caenorhabditis elegans* CSP orthologue, *dnj-14*, to identify which tissues preferentially express CSP and hence may contribute to the observed mutant phenotypes. Replacement of *dnj-14* with wrmScarlet caused a strong chemotaxis defect, as seen with other *dnj-14* null mutants. In contrast, inserting the reporter in-frame to create a DNJ-14-wrmScarlet fusion protein had no effect on chemotaxis, indicating that C-terminal tagging does not impair DNJ-14 function. WrmScarlet fluorescence appeared most obvious in the intestine, head/pharynx, spermathecae and vulva/uterus in the reporter strains, suggesting that DNJ-14 is preferentially expressed in these tissues. Crossing the DNJ-14-wrmScarlet strain with GFP marker strains confirmed the intestinal and pharyngeal expression, but only a partial overlap with neuronal GFP was observed. DNJ-14-wrmScarlet fluorescence in the intestine was increased in response to starvation, which may be relevant to mammalian CSPα’s role in microautophagy. DNJ-14’s enrichment in worm reproductive tissues (spermathecae and vulva/uterus) parallels the testis-specific expression of CSPβ and CSPγ isoforms in mammals. Furthermore, CSPα messenger RNA is highly expressed in the human proximal digestive tract, suggesting that CSP may have a conserved, but overlooked, function within the gastrointestinal system.

**Supplementary Information:**

The online version contains supplementary material available at 10.1007/s00441-024-03875-w.

## Introduction

Cysteine string protein (CSP) is a member of the DnaJ/Hsp40 family of co-chaperones. It is named after a characteristic string of cysteine residues, palmitoylation of which is required for CSP’s membrane localisation and post-Golgi trafficking (Greaves and Chamberlain 2006; Greaves et al. 2008). Mammalian genomes contain three CSP-encoding genes: DNAJC5, DNAJC5B and DNAJC5G, which encode CSPα, CSPβ and CSPγ, respectively, whereas invertebrates such as *Drosophila* and *Caenorhabditis elegans* have only one CSP-encoding gene. CSP is widely accepted to use its molecular chaperone function to prevent protein misfolding, most notably of the SNARE protein SNAP-25 (Chandra et al. 2005; Sharma et al. 2011). However, in recent years, CSP has been shown to also function in two different proteostasis pathways: misfolding-associated protein secretion (MAPS) (Deng et al. 2017; Fontaine et al. 2016; Lee et al. 2022, 2018) and endolysosomal microautophagy (Lee et al. 2022). Thus, CSP is thought to counteract the toxic effects of intracellular misfolded proteins, by targeting them for either refolding, secretion or degradation. Mutations in CSP-encoding genes in *Drosophila* (Imler et al. 2019; Zinsmaier et al. 1994), *C. elegans* (Barker et al. 2023; Kashyap et al. 2014), mice (Fernandez-Chacon et al. 2004) and humans (Benitez et al. 2011; Noskova et al. 2011) result in neuronal dysfunction, neurodegeneration and reduced lifespan. CSP therefore has an evolutionarily conserved neuroprotective function(s).

CSP was originally discovered in *Drosophila* using a monoclonal antibody that preferentially labelled neurons (Zinsmaier et al. 1990). CSP immunoreactivity was observed in all neuropil regions and in synaptic boutons of motor neurons. Within the same study, in situ hybridisation also revealed CSP expression in the retina and neuronal cell bodies (Zinsmaier et al. 1990). A follow-up study identified a more widespread distribution of CSP in *Drosophila* tissues using immunohistochemistry. CSP was found in high levels in all synaptic terminals, ovarian follicular cells, tall cells of the cardia and specific regions of the male reproductive tract, in addition to low levels throughout all tissues examined (Eberle et al. 1998). An independent investigation into synaptic protein localisation in *Drosophila* photoreceptor terminals also identified CSP expression in the lamina, in addition to the medulla and central brain neuropils (Hamanaka and Meinertzhagen 2010).

CSPα tissue distribution has also been assessed in rats, using anti-CSPα antibodies. CSPα expression was particularly high in synapse-rich regions of the brain, specifically that of the cerebellum, retina, hippocampal formation and main olfactory bulb (Kohan et al. 1995). Prominent CSPα immunoreactivity was also observed in chromaffin cells of the rat adrenal medulla (Kohan et al. 1995). Indeed, CSPα was found to be expressed in a range of non-neuronal rat tissues including the liver, kidney, spleen, lung and adrenal gland through PCR, and validated by Northern blotting (Chamberlain and Burgoyne 1996). These findings of widespread distribution of CSPα across tissues were replicated in human tissues through Northern blot analysis, detecting CSPα in the liver, kidney, lung, pancreas, brain, heart, placenta and skeletal muscle (Coppola and Gundersen 1996). CSPα has also been identified in enterochromaffin-like cells in the rat stomach (Zhao et al. 1997) and in pinealocytes within the gerbil pineal gland (Redecker et al. 1998).

In neurons, CSPα is mainly localised to synaptic vesicles within the presynaptic terminal (Mastrogiacomo et al. 1994; Zinsmaier et al. 1994). A fraction of neuronal CSPα also co-localises with lysosomal markers in the soma, neurites and synaptic boutons (Benitez and Sands 2017). In non-neuronal cells, CSPα has been associated with a range of regulated secretory organelles such as insulin-containing granules of β-cells of the pancreas (Brown et al. 1998), the membranes of pancreatic zymogen granules (Braun and Scheller 1995) and chromaffin granules of the adrenal medulla (Chamberlain et al. 1996). CSPα has also been associated with late endosomes and lysosomes in its role in MAPS, where CSPα was identified to facilitate the extracellular export of proteins enriched on the surface of the endoplasmic reticulum (ER), and thus has also been linked to the ER and the cell surface (Lee et al. 2022; Xu et al. 2018). Indeed, CSPα has been associated with the ER in its role in regulating the exit of CFTR (Schmidt et al. 2009). CSPα was additionally identified in a proteomic screen of proteins associated with the autophagosome (Dengjel et al. 2012). Finally, CSPα has been shown to localise to the plasma membrane in adipocytes, where it functions in the insulin-dependent fusion of Glut4 storage vesicles with the plasma membrane (Chamberlain et al. 2001).

CSP’s function in *C. elegans* has been explored by mutational analyses of its orthologue *dnj-14* (Barker et al. 2023; Chen et al. 2015; Kashyap et al. 2014; McCue et al. 2015; Mulcahy et al. 2019). However, it remains unknown which worm cells/tissues express DNJ-14 protein and hence may contribute to *dnj-14* mutant phenotypes. We therefore set out to perform an unbiased analysis of CSP expression in *C. elegans* by using genome editing to fluorescently tag *dnj-14* in its natural chromosomal locus. The transparent nature of *C. elegans*, combined with its simple anatomy comprising only 959 cells, allows for fluorescent labelling and visualisation of proteins in all cells simultaneously in vivo (Li and Le 2013), which is not possible in *Drosophila* and mammalian models. Analysing protein expression in vivo also enables assessment of dynamic protein expression, such as with age or following treatment with stressors or drugs. Furthermore, *C. elegans* can also easily be crossed onto strains expressing fluorescent reporters driven by tissue-specific promoters, to allow for assessment of co-localisation and therefore validating tissue distribution. To label endogenous CSP, the wrmScarlet fluorescent protein tag was chosen. WrmScarlet is a *C. elegans* codon-optimised version of mScarlet, a bright monomeric red fluorescent protein (RFP) (Redemann et al. 2011). Given that most *C. elegans* tissue-specific marker strains express green fluorescent protein (GFP), tagging *dnj-14* with RFP facilitates assessment of co-localisation following crossing with these GFP marker strains. Compared with other RFPs, mScarlet outperforms in terms of cytotoxicity, fluorescence lifetime and brightness analysis (Bindels et al. 2017). Indeed, wrmScarlet has an eightfold increase in fluorescence compared with TagRFP-T (El Mouridi et al. 2017), which should allow for detection of endogenous DNJ-14 even in areas of relatively low expression. Here, we report the use of the wrmScarlet reporter to map DNJ-14 tissue distribution in *C. elegans*, revealing preferential expression in the intestine, head/pharynx, spermatheca and vulva/uterus.

## Materials and methods

### Nematode culture

*C. elegans* were grown and cultured at 20 °C on nematode growth media (NGM; 2% (w/v) agar, 0.3% (w/v) NaCl, 0.25% (w/v) peptone, 1 mM CaCl_2_, 1 mM MgSO_4_, 25 mM KH_2_PO_4_, 5 µg/mL cholesterol). *Escherichia coli* OP50 supplemented with 50 µg/mL kanamycin was used as a food source. NGM plates were occasionally supplemented with 50 µg/mL kanamycin and 100 units/mL nystatin for cleaning; after validating it did not interfere with reported phenotypes. Bristol N2 was used as the wild-type reference strain. A complete list of *C. elegans* strains used in this study is provided in Table 1.

**Table 1:** List of *C. elegans* strains used in this study

Strain name	Allele	Description	Source/reference
N2	Wild-type	Wild-type	Magnitude Biosciences (UK)
*dnj-14(null)*	*ulv20*	Deletion of 1208 bp from the *dnj-14* open reading frame (created by CRISPR and repaired by non-homologous end-joining)	(Barker et al. 2023)
*dnj-14(wrmScarlet null)*	*syb4033*	Precise replacement of the entire open reading frame of *dnj-14* with wrmScarlet (created by CRISPR and repaired by homologous recombination)	This study (microinjections carried out by SunyBiotech (China))
*dnj-14(wrmScarlet fusion)*	*syb4338*	Insertion of wrmScarlet in-frame after the final *dnj-14* codon to make a C-terminal fusion protein (created by CRISPR and repaired by homologous recombination)	
KN259	*huIs33*	*sod-3::gfp* + *rol-6(su1006)* GFP expressed under the control of the *sod-3* promoter Encodes mutant collagen *(rol-6(su1006))* that induces a dominant ‘roller’ phenotype	Caenorhabditis Genetics Center (USA)
SJ4005	*zcls4*	*Phsp-4::gfp* GFP expressed under the control of the *hsp-4* promoter	
NM2415	*jsls68*	*Prab-3::gfp-rab3* GFP-tagged RAB-3 expressed under the control of the *rab-3* promoter	

### *C. elegans* strain construction

Homozygous *dnj-14* wrmScarlet strains were created by CRISPR/Cas9 genome editing. The *dnj-14(wrmScarlet null)* strain was generated utilising one single guide RNA (sgRNA) targeting the 5′ end of the first exon of *dnj-14* (TTCAGGGAAATGAACTCAGACGG) and two sgRNAs targeting the 3′ end of the last exon of *dnj-14* (CCGATTGTGATTGCCATGCCTCC and CACCGCCTTCTCAAAAGTATGGG) to produce a clean deletion of the open reading frame. The *dnj-14(wrmScarlet fusion)* allele was generated using the same two sgRNAs targeting the 3′ end of the last exon of *dnj-14* described above to produce an in-frame insertion at the C-terminus of the DNJ-14 protein. The DNA double-strand breaks induced by Cas9 were repaired by homologous recombination with complementary DNA (cDNA) encoding wrmScarlet (El Mouridi et al. 2017) flanked by sequence corresponding to the 5′ and 3′ ends of the *dnj-14* open reading frame for the *dnj-14(wrmScarlet null)* allele, and just the 3′ end for the *dnj-14(wrmScarlet fusion)* allele. Gonadal injections of recombinant purified Cas9, sgRNAs and cDNAs and selection of edited worm lines were performed by SunyBiotech (China). Inheritance of the *wrmScarlet* allele was verified through PCR primers (forward: 5′-TCTCCCAATTTTCGCGCTCT-3′; reverse: 5′-AGGGGGAGAAAAGGGGAGAA-3′) which produce differentially sized products for WT and *dnj-14* wrmScarlet null and fusion alleles (WT, 1792 bp; *dnj-14(wrmScarlet null)*, 1008 bp; *dnj-14(wrmScarlet fusion)*, 2485 bp). All *dnj-14* mutant strains were further validated by sequencing (DNA Sequencing and Services, University of Dundee, UK).

### Crossing of dnj-14(wrmScarlet fusion) *C. elegans* with translational GFP neuronal reporter strain

*dnj-14(wrmScarlet fusion)* hermaphrodites were initially outcrossed with N2 males. The resulting males from this cross were further crossed onto NM2415 (*Prab-3::rab-3::GFP*) hermaphrodites, and offspring were selected for both green and red progeny in each generation, until there was no non-fluorescent progeny visible.

### Crossing of dnj-14(wrmScarlet fusion) *C. elegans* with transcriptional GFP intestinal reporter strains

N2 males were initially crossed with *dnj-14(wrmScarlet fusion)* hermaphrodites. The resulting males from this cross were further crossed onto either KN259 *(huIs33 [Psod-3::GFP; rol-6(su1006)])* or SJ4005 *(zcIs4 [Phsp-4::GFP])* worms and selected for both green and red progeny in each generation, until there was no non-fluorescent progeny visible. Crosses involving the *Psod-3::GFP; rol-6(su1006)* strain additionally allowed for selection of worms with a ‘roller’ phenotype, conferred by a dominant mutation in the collagen-encoding gene *rol-6*.

### Age synchronisation

NGM plates containing gravid worms were washed with 3.5 mL sterile H_2_O and added to 1 mL commercial bleach and 0.5 mL of 5 M NaOH. Following vortexing every 2 min for 10 min, the sample was centrifuged at 1000 g for 1 min to pellet the released eggs. The supernatant was removed, and the pellet washed with 5 mL sterile H_2_O to remove any residual bleach. The suspension was again centrifuged at 1000 g for a further minute, prior to aspirating the supernatant. The resultant pellet was resuspended in 100 µL sterile H_2_O and pipetted onto the edge of a newly seeded NGM plate.

### Food race assays

Age-synchronised adult day 1–3 worms were washed twice for 15 min through placing in M9 buffer (22 mM KH_2_PO_4_, 42 mM Na_2_·HPO_4_, 85.5 mM NaCl, 1 mM MgSO_4_) with 0.1% (w/v) bovine serum albumin (BSA) and allowing to thrash, both to remove residual OP50 on the surface of the animals and to allow time for starvation behaviours to commence. Washed worms were placed 30 mm away from a 30-µL droplet of OP50, seeded 48 h previously. The number of animals reaching the food was recorded every 10 min for 120 min. A minimum of 30 worms per strain were assayed for each timepoint tested, from at least three independent experimental repeats. Statistical analysis was performed using a log-rank test. An error probability level of *P* < 0.05 was accepted as statistically significant; however, exact *P* values for each statistical test are indicated in each figure and figure legend.

### Starvation stress

Age-synchronised worms were reared on NGM plates seeded either with sufficient OP50 bacterial food to last > 3 days (fed worms) or with a small amount of OP50 that was rapidly consumed, causing the worms to starve. For starvation time courses, worms were transferred to unseeded NGM plates containing no bacteria for 2–24 h and compared to worms grown on NGM seeded with 200 µL of OP50 as a control. Worms were imaged at the adult day 3 stage.

### Heat stress

Adult day 1 worms were heated to 35 °C for 2 h on NGM plates seeded with OP50, followed by recovery at 20 °C for 24 h prior to imaging, using worms grown at 20 °C as a control.

### Osmotic stress

Adult day 1 worms were subjected to ethanol concentrations ranging from 0 to 2 M for 2 h in M9 buffer containing 0.1% (w/v) BSA and imaged after 24 h.

### *C. elegans* imaging

Live worms were placed in a droplet of M9 buffer containing 1% (v/v) 30-µm-diameter polystyrene microbeads (to prevent crushing by coverslips) and 1 mg/mL levamisole (to immobilise worms) on a Superfrost Plus™ microscope slide. A cover slip was placed on top of the nematodes and sealed with nail varnish. The worms were imaged immediately either on a Nikon Eclipse Ti inverted fluorescence microscope, using NIS-Elements imaging software, or on a Leica DMi8 Andor Dragonfly multi-point confocal microscope system, using the inbuilt Andor imaging software. Supplementary Table 1 summarises microscope configurations and image acquisition parameters utilised for images taken with the Dragonfly system.

## Results

### Generation of *C. elegans* DNJ-14 fluorescent reporters

**Fig. 1:**
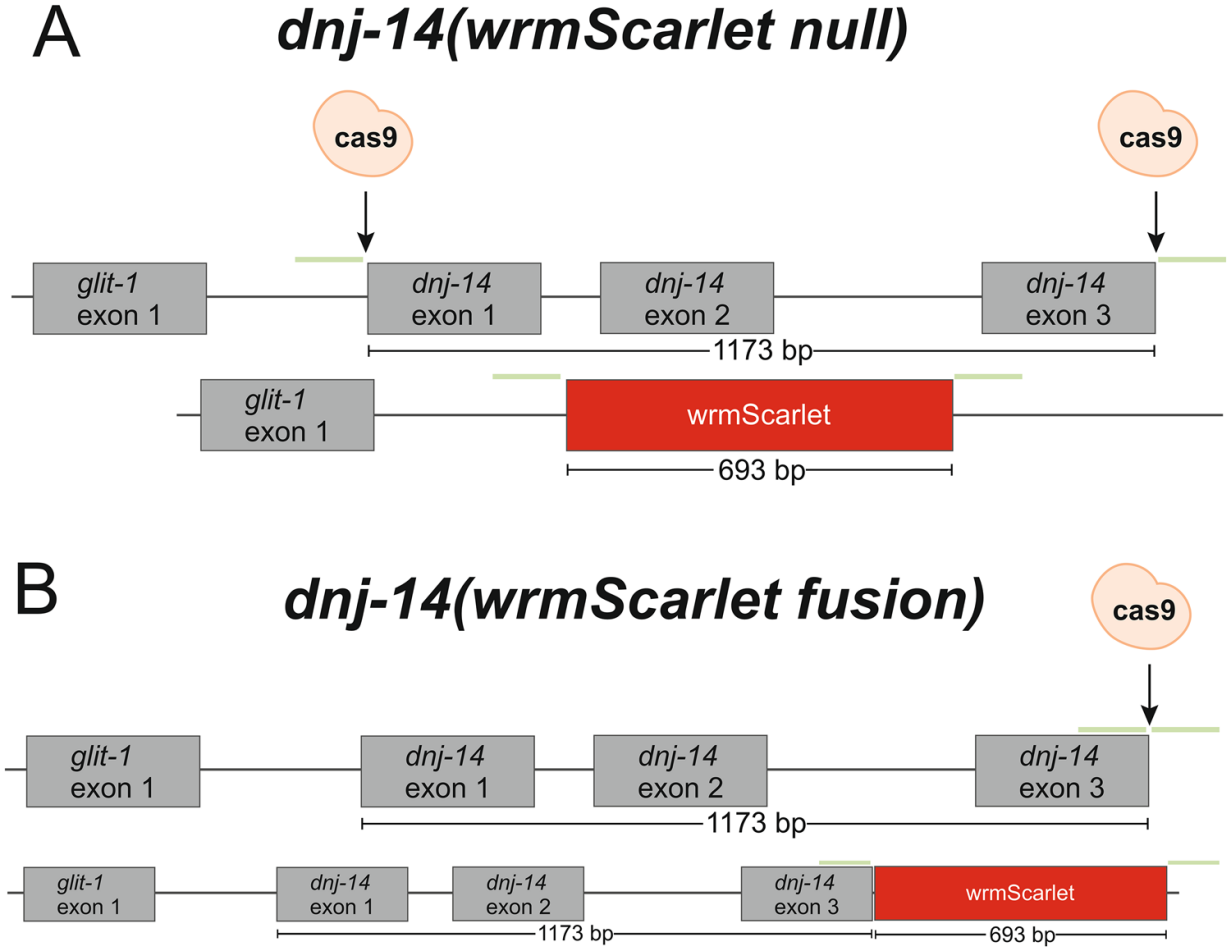
CRISPR-Cas9 strategy for generating *dnj-14* wrmScarlet null and fusion alleles. Relative positions of Cas9 cut sites for *dnj-14(wrmScarlet null)* (**A**) and *dnj-14(wrmScarlet fusion)* (**B**) alleles are shown. Schematic diagrams of the insertion following successful genome editing are indicated below. Homology arms used in each repair template are complimentary to the regions indicated in green

The *C. elegans* genome encodes a single orthologue of the *DNAJC5* gene encoding CSP, *dnj-14*. In order to investigate the tissue localisation of DNJ-14 in *C. elegans*, we used CRISPR/Cas9 to introduce fluorescent reporters into the endogenous *dnj-14* chromosomal locus. Two such reporter strains were created: (1) a transcriptional reporter that simultaneously deleted the entire *dnj-14* open reading frame (ORF) to produce a null allele expressing free wrmScarlet protein under control of the dnj-14 promoter and (2) a C-terminal reporter inserted in-frame to preserve expression of DNJ-14 as a wrmScarlet-tagged fusion protein. To generate the null transcriptional fluorescent reporter, sgRNAs targeting the 5′ and 3′ ends of the *dnj-14* ORF were used, thereby directing the Cas9 cut sites to excise the intervening sequence (Fig. 1A). Co-injection of cDNA encoding the wrmScarlet fluorescent reporter flanked by homology arms enabled its insertion in place of *dnj-14* at the endogenous locus, hereafter referred to as *dnj-14(wrmScarlet null)*. To eliminate concerns that removal of *dnj-14* itself may in turn alter expression of wrmScarlet, a second strain was generated whereby wrmScarlet was tagged to the C-terminus of endogenous *dnj-14*, hereafter referred to as *dnj-14(wrmScarlet fusion)*. This was achieved using sgRNA targeting the C-terminus of *dnj-14* (Fig. 1B). We reasoned that tagging the C-terminus would be unlikely to alter endogenous CSP localisation and function, as GFP tagging of the C-terminus of mammalian CSPα does not alter CSPα localisation (Barker et al. 2024; Sharma et al. 2011). Both *dnj-14(wrmScarlet null)* and *dnj-14(wrmScarlet fusion)* alleles were repaired with wrmScarlet cDNA flanked by homology arms complimentary to the regions adjacent to the desired insertion sites (Fig. 1B). Gene-edited *dnj-14(wrmScarlet null)* and *dnj-14(wrmScarlet fusion)* lines were identified initially through observations of red fluorescence, followed by PCR genotyping (Supplementary Figs. S1A, S2A, respectively) and finally validated through sequencing (Supplementary Figs. S1B, S2B, respectively).

**Fig. 2:**
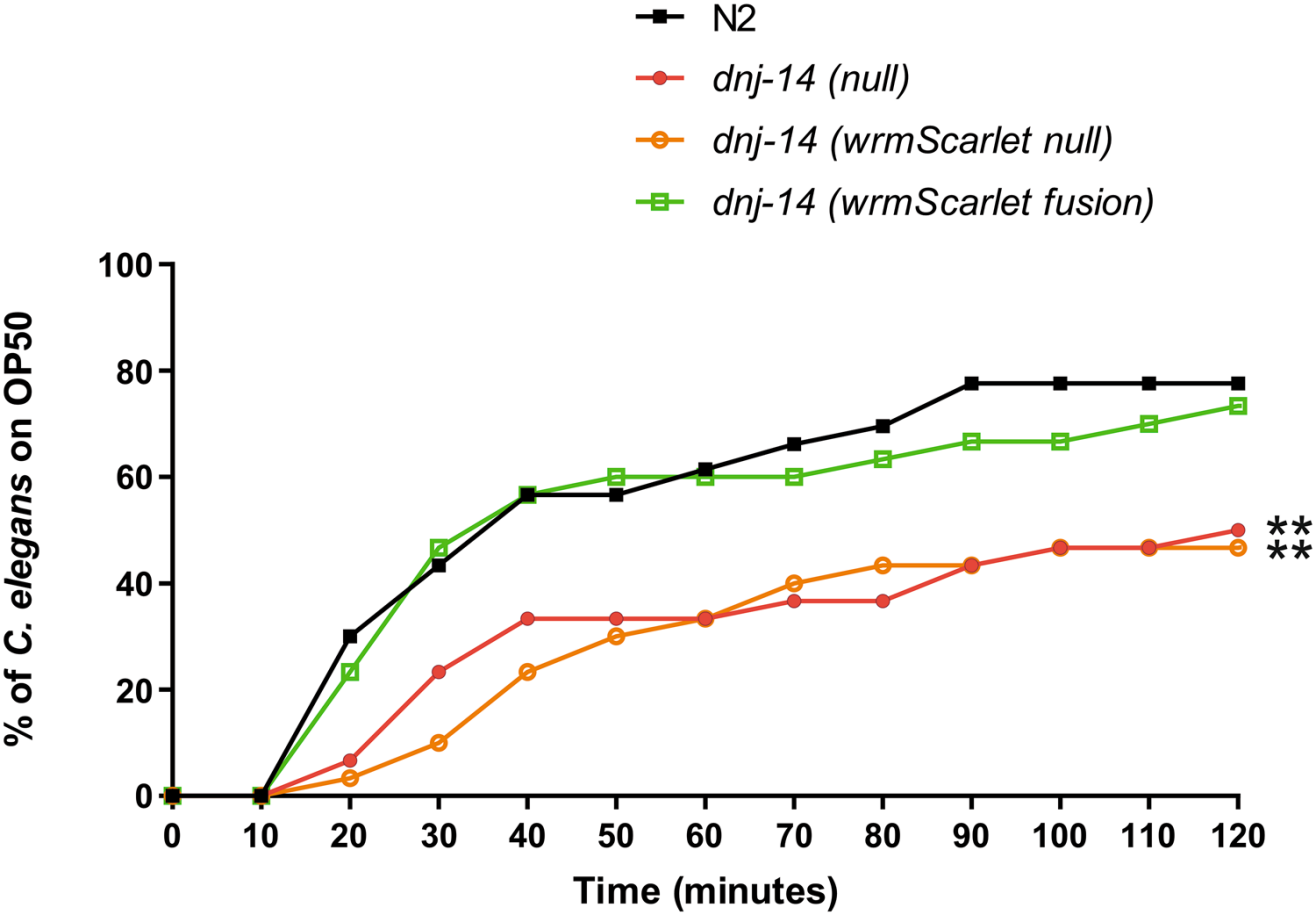
WrmScarlet tag does not interfere with *dnj-14* function. Chemotaxis defects were assayed through measuring the time taken for worms to reach a bacterial food source 30 mm away. *C. elegans* were age-synchronised and assayed between 1 and 3 days of adulthood. A minimum of 30 worms were assayed per strain from three independent biological repeats. *dnj-14(null)* and *dnj-14(wrmScarlet null)* have significantly impaired chemotaxis compared with N2 WT controls. **(*P* < 0.01). *dnj-14(wrmScarlet fusion)* worms do not have a significant defect in chemotaxis compared to the N2 WT controls. The chemotaxis defect in *dnj-14(wrmScarlet null)* worms was not significantly different from that of *dnj-14(null)* worms

To establish whether the wrmScarlet tag itself interfered with CSP function, a chemotaxis assay was utilised, whereby the time taken for worms to reach an attractive bacterial food source is assessed. This assay was chosen because it has been previously reported to be a strong and reproducible phenotype induced by various *dnj-14* mutants (Barker et al. 2023; Chen et al. 2015; Kashyap et al. 2014). Consistent with this, a significant (*P* < 0.01) defect in chemotaxis was observed in the previously described *dnj-14(null)* worm strain (Barker et al. 2023). Similarly, our newly created *dnj-14(wrmScarlet null)* mutant exhibited a significant defect in chemotaxis compared with N2 controls (*P* < 0.01). Importantly, the *dnj-14(wrmScarlet null)* mutant was not significantly different to the *dnj-14(null)*, nor were *dnj-14(wrmScarlet fusion)* worms significantly different to N2 controls (Fig. 2). Altogether, this indicates that insertion of a C-terminal wrmScarlet tag does not interfere with *dnj-14* function and hence it could be used to probe the tissue distribution of the endogenous DNJ-14 protein.

### DNJ-14 appears to be expressed predominantly in the digestive and reproductive tracts

**Fig. 3:**
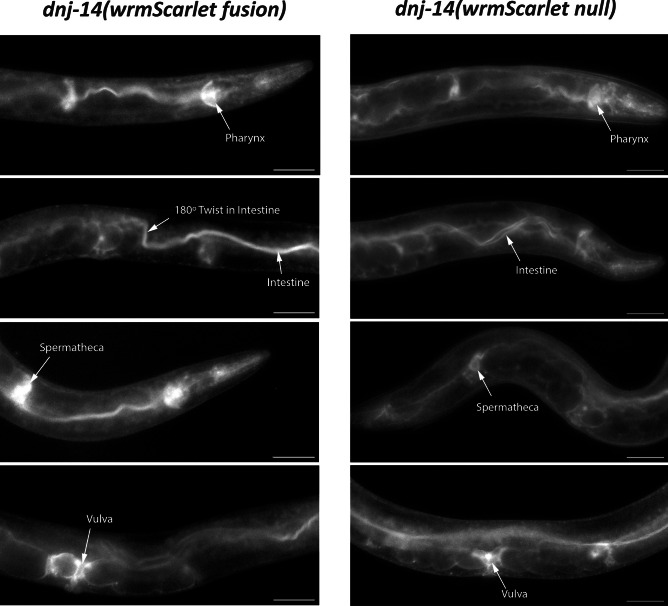
DNJ-14 is primarily expressed in *C. elegans* digestive and reproductive tissues. Representative images of *dnj-14(wrmScarlet fusion)* (left panel) and *dnj-14(wrmScarlet null)* (right panel) worms are shown. Images were acquired on a Nikon Eclipse Ti inverted fluorescence microscope, with NIS-Elements microscope imaging software with a × 20 objective lens. Scale bars, 50 µm

Having created both the *dnj-14* transcriptional reporter and C-terminal translational fusion and established that the wrmScarlet tag did not appear to interfere with *dnj-14* function, the expression pattern of wrmScarlet was analysed through fluorescence microscopy. The most obvious expression was observed in the head and in a long projection running the length of the worm (Fig. 3). Given that this projection connected anteriorly to the pharynx and displayed the characteristic 180° twist that occurs in the intestine at the longitudinal body axis (Fig. 3), it is likely to be the intestine. The DNJ-14 expression occurring within the head appeared to be mainly in the pharynx, as the observed morphology followed the ‘two bulb’ structure of the pharynx, with a connecting isthmus (Fig. 3). The expression could also potentially be occurring in IL2 chemosensory neurons which surround the pharynx. However, as these projections originate at the isthmus of the pharynx and terminate at the anterior surface of the worm, these do not cover the posterior bulb of the pharynx where wrmScarlet fluorescence was also observed. Thus, DNJ-14 expression in the head cannot be solely explained by expression in IL2 neurons. Two large punctae of fluorescence were also observed within the body (Fig. 3), whose position lateral to the embryos along the worm’s lateral axis suggested that these could be spermathecae, as *C. elegans* have two spermathecae, one at the end of each gonad. The expression occurring in the midline appeared to be in vulval muscles and the uterine epithelia, based on the position medial to the developing embryos and adjacent to the vulval opening.

**Fig. 4:**
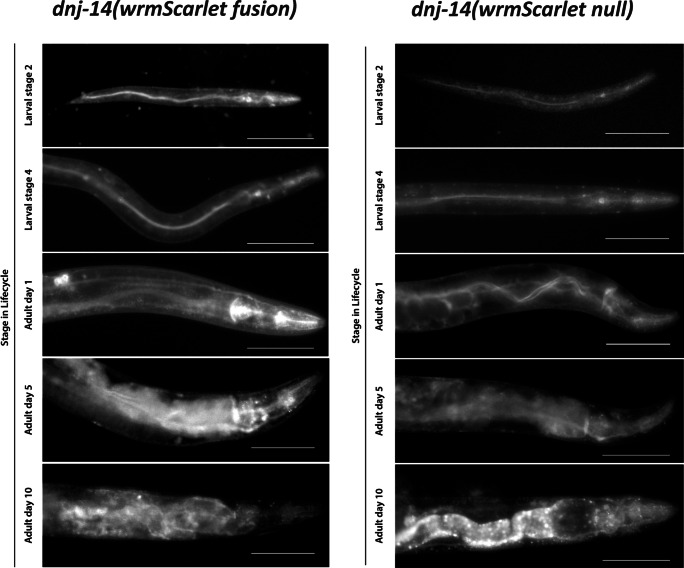
DNJ-14 expression throughout the *C. elegans* lifespan. Representative images of *dnj-14(wrmScarlet fusion)* (left panel) and *dnj-14(wrmScarlet null)* (right panel) worms are shown, from the larval stage, until day 10 of adulthood. Images were acquired on a Nikon Eclipse Ti inverted fluorescence microscope, with NIS-Elements microscope imaging software, with a × 20 objective lens. Scale bars, 100 µm

The expression patterns observed in *dnj-14(wrmScarlet null)* and *dnj-14(wrmScarlet fusion)* worms were similar (Fig. 3). Both displayed strong expression in the head/pharynx, the intestine, the vulva/uterus and spermathecae. Whilst imaging the worms, it was noted that the expression patterns altered depending on the stage of the *C. elegans* lifecycle. Therefore, we next age-synchronised both strains and imaged the worms over several stages of their lifespan, from larvae to day 10 of adulthood (Fig. 4). For both alleles, up until day 1 of adulthood, the prominent features were the head/pharynx and intestine. Strong diffuse expression appeared around day 5 of adulthood, which obscured other structures within the worm. By day 10 of adulthood, expression in the intestine was extremely bright in the *dnj-14(wrmScarlet null)* (Fig. 4). This was also the case to an extent with the *dnj-14(wrmScarlet fusion)*, although it was somewhat obscured by diffuse fluorescence, which may be due to age-induced lipofuscin accumulation (Fig. 4).

### dnj-14 expression alters upon starvation-induced stress

**Fig. 5:**
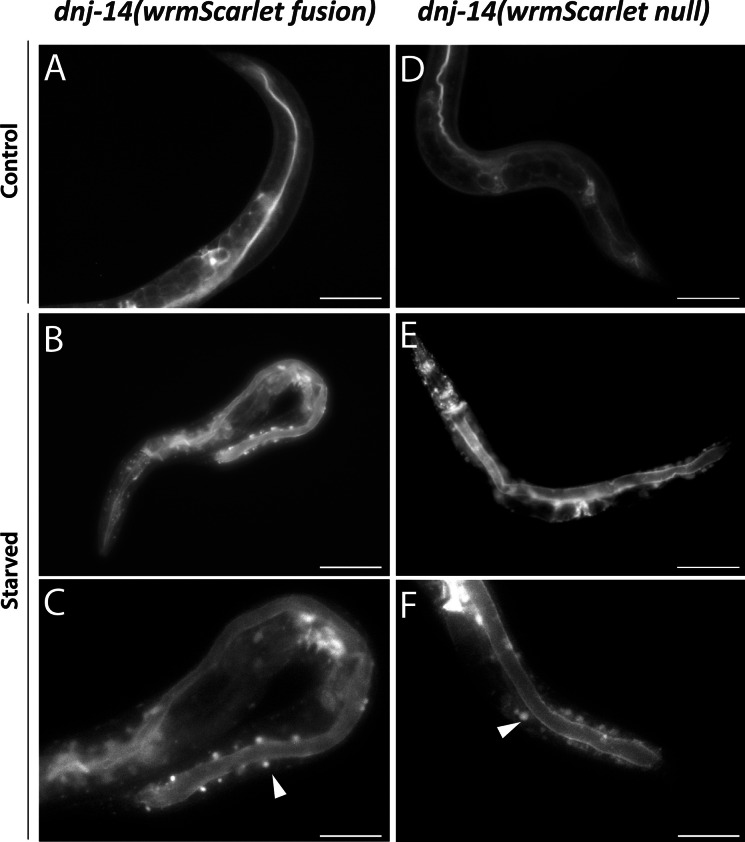
DNJ-14 exhibits differential expression patterns upon food deprivation. Compared here are representative images of *dnj-14(wrmScarlet fusion)* (left panel) and *dnj-14(wrmScarlet null)* (right panel) worms. *C. elegans* were grown on standard NGM in the presence of either copious amounts of OP50 (**A**, **D**) or a very small quantity of OP50 which was quickly depleted, leaving the worms to starve (**B**–**F**). Images were acquired on a Nikon Eclipse Ti inverted fluorescence microscope, with NIS-Elements microscope imaging software, using × 20 (**A**, **B**, **D**, **E**) and × 40 (**C**, **F**) objective lenses. Scale bars, 100 µm (**A**, **B**, **D**, **E**) and 50 µm (**C**, **F**)

Whilst acquiring these images under normal conditions, it was noted that there were differential expression patterns of DNJ-14 depending on whether the worms being imaged were obtained from a well-fed NGM plate, or one that was becoming depleted of the OP50 bacterial food source. Therefore, we next compared age-synchronised worms that were well fed with copious quantities of OP50 with those that were given a small quantity of OP50 which was quickly depleted, leaving the worms to starve over the following days. Consistent with our initial observations, both the *dnj-14(wrmScarlet null)* and *dnj-14(wrmScarlet fusion)* displayed differential expression profiles dependent on whether the worms were fed or starved. DNJ-14 expression in the fed worms appeared to mainly increase within the intestinal lumen, due to its thin appearance and localisation in the centre of the intestine. In contrast, the starved worms exhibited increased expression across the intestine, in addition to bright punctate structures clustered around the periphery of the intestinal tract, which were primarily present on the posterior half of the worm (Fig. 5). Next, to establish whether this change in expression occurred rapidly upon removal from food sources, as changes in gene expression and metabolism can occur within minutes in *C. elegans* (Baugh and Hu 2020), a starvation time course was performed. Worms were removed entirely from their bacterial food source and imaged at set time intervals between 2 and 20 h. The food-deprived worms appear to display similar expression patterns to the fed worms until 6 h of food deprivation. At 20 h of food deprivation, the increased expression across the intestine and expression in bright puncta surrounding the intestine can be observed, suggesting this expression change occurs somewhere between 6 and 20 h of food deprivation (Supplementary Fig. S3).

Having established that food deprivation alters DNJ-14 expression, we then sought to determine whether this change in expression is triggered by a general stress response. To assess this, both the *dnj-14(wrmScarlet null)* and *dnj-14(wrmScarlet fusion)* worms were subjected to heat shocking at 35 °C. However, no change in wrmScarlet expression was observed between the control and heat-shocked condition with either the transcriptional null or the C-terminal fusion fluorescent reporters (Supplementary Fig. S4). Changes in expression following osmotic stress were then tested through exposure to high ethanol concentrations. However, no change in wrmScarlet expression was observed in either the transcriptional null or the C-terminal fusion fluorescent reporters between 0 and 2 M ethanol (Supplementary Fig. S5). Together, this suggested that changes in DNJ-14 expression are induced specifically by food deprivation, and not through a general stress response pathway.

### DNJ-14 co-localises with intestinal markers

**Fig. 6:**
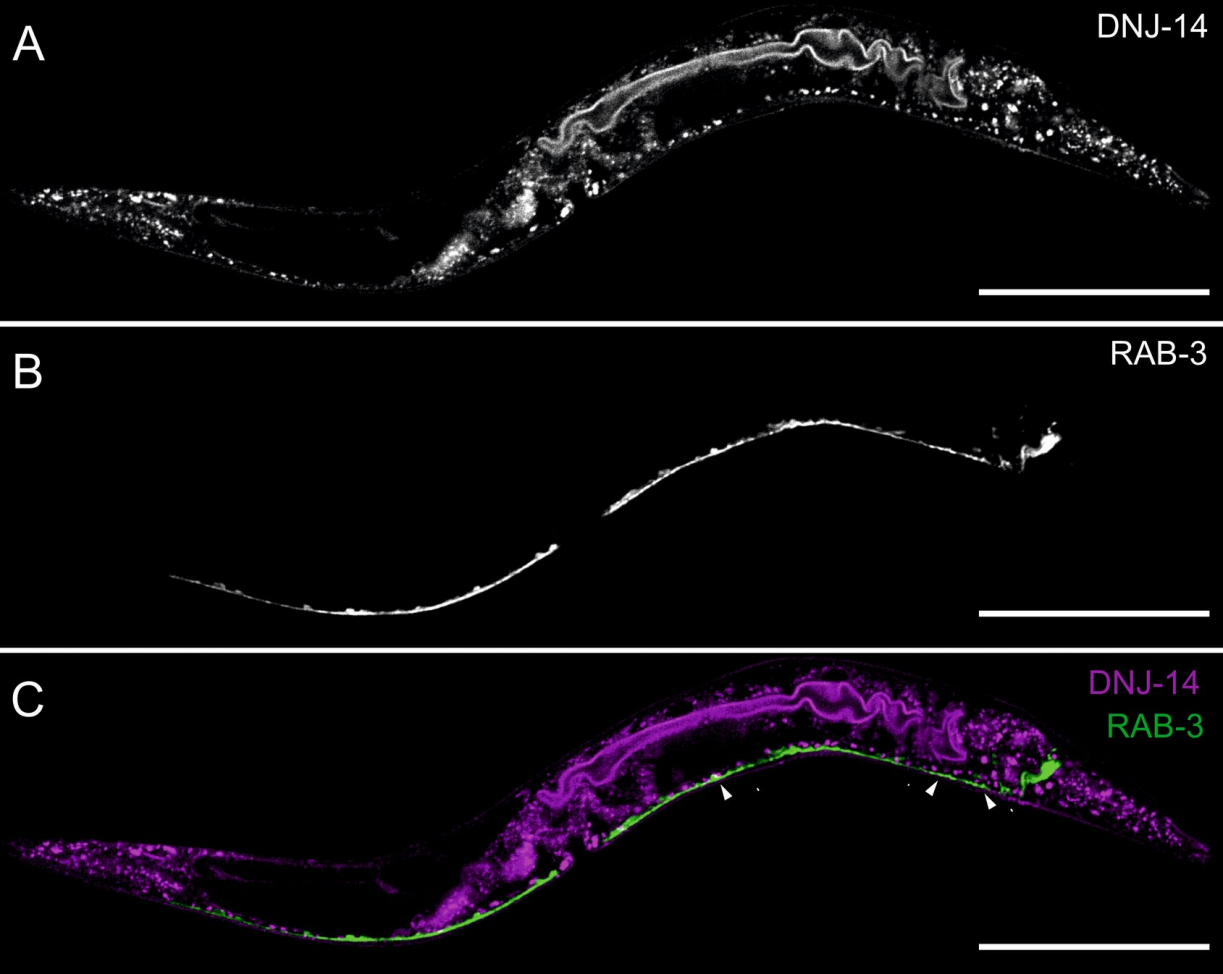
DNJ-14::wrmScarlet exhibits only a small overlap with neuronal RAB-3::GFP. Representative images using 561 nm excitation of *dnj-14(wrmScarlet)* (**A**) and 488 nm excitation of *Prab-3::GFP-rab-3* (**B**) in double-transgenic worms. Merged images of **A** and **B** are shown in **C**, with regions of co-localisation indicated by arrowheads. Images were acquired on an Andor Dragonfly confocal system at × 40 magnification. Scale bars, 100 µm

Given the documented neuronal expression of CSP in flies and CSPα in mammals, we sought to determine the extent of DNJ-14 expression in *C. elegans* neurons. RAB-3 is a synaptic vesicle protein that is enriched in the worm nervous system, notably in the nerve ring and ventral and dorsal nerve cord regions (Cheng et al. 2015). RAB-3 localisation was determined using worms expressing GFP-tagged RAB-3 driven by a *rab-3* promoter, which is widely used as a neuronal reporter. This pan-neuronal marker strain was crossed onto our *dnj-14(wrmScarlet fusion)* allele, and the resultant double-transgenic reporter worms were then assessed for co-localisation of fluorophores (Fig. 6). Fluorescence was observed using an Andor Dragonfly multi-point confocal system to gain increased resolution of *C. elegans* tissues. It was evident through comparing expression patterns that there was only a relatively small degree of co-localisation of DNJ-14 and RAB-3. The regions of co-localisation were small punctate signals of DNJ-14 which overlap with parts of the ventral nerve cord labelled by RAB-3, possibly representing neuromuscular junctions and neuronal cell bodies. However, the majority of DNJ-14 expression clearly did not co-localise with RAB-3.

**Fig. 7:**
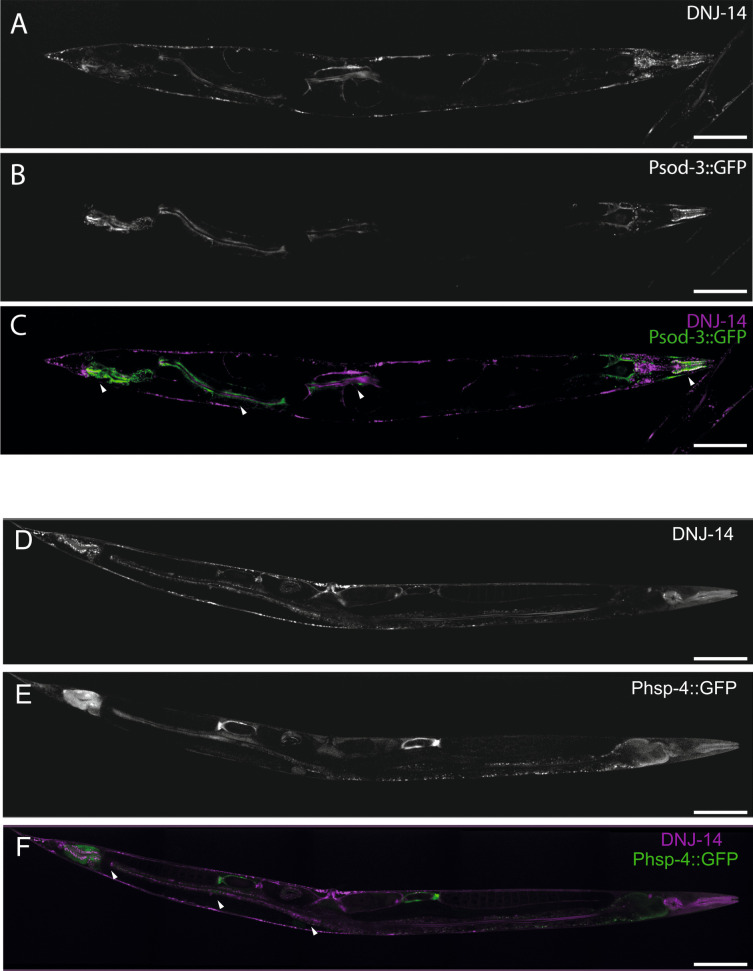
DNJ-14::wrmScarlet partially co-localises with GFP driven by *sod-3* and *hsp-4* promoters. Representative images using 561 nm (**A**, **D**) and 488 nm (**B**, **E**) excitation of *dnj-14(wrmScarlet); Psod-3::GFP* and *dnj-14(wrmScarlet); Phsp-4::GFP* double-transgenic worms. Merged images are shown in **C** and **F**, with regions of co-localisation indicated by arrowheads. Images were acquired on an Andor Dragonfly confocal system at × 40 magnification. Scale bars, 100 µm

Having determined that much of DNJ-14 expression was occurring outside of the nervous system, to identify if this was indeed intestinal as originally suspected, the *dnj-14(wrmScarlet fusion)* strain was crossed onto worms expressing GFP intestinal reporters. As the available *C. elegans* intestinal reporters are not entirely specific to the intestine, two intestinal reporters were utilised: *Psod-3::GFP* and *Phsp4::GFP*. *Psod-3::GFP* is expressed in both the intestine and pharynx, and *Phsp-4::GFP* is expressed in the intestine and other tissues, including the hypodermis (MacNeil et al. 2015). This was achieved by crossing *dnj-14(wrmScarlet fusion)* mutants onto worms expressing GFP under either *sod-3* or *hsp-4* promoters and selecting for both green and red progeny in each generation until homozygosity was reached. Given that neither of these intestinal markers are entirely specific to the intestine and that DNJ-14 expression does not exclusively occur within the intestine, complete overlap of fluorescent signals was not expected. When comparing the fluorescent signals, there was clear overlap between wrmScarlet and GFP expressed under both the *sod-3* and *hsp-4* promoters, suggesting that DNJ-14 is indeed expressed within the intestine (Fig. 7). As *Psod-3::GFP* is also expressed within the pharynx, validation of pharyngeal localisation of DNJ-14 was additionally confirmed (indicated by an arrowhead on Fig. 7C).

The observation of high non-neuronal expression of CSP was a little surprising, given that most studies of mammalian CSPα have focused on its role in neurons. Thus, our findings in *C. elegans* were compared to those in humans from a non-biased approach to assess the relevance of these findings to humans, using the Human Protein Atlas (HPA). The HPA provides a complete map of protein expression across 32 human tissues. CSPβ and CSPγ have high tissue specificity and are mainly restricted to the testis, whereas CSPα expression has low tissue specificity and is detected in all tissues tested (Uhlén et al. 2015). Although CSPα messenger RNA (mRNA) is most strongly expressed in neural tissues, the second highest expression of CSPα in humans is actually in the proximal digestive tract, especially in the oesophagus (https://www.proteinatlas.org/ENSG00000101152-DNAJC5/tissue), which corresponds with the strong expression of DNJ-14 observed here within the *C. elegans* intestinal tract.

## Discussion

In this study, two DNJ-14 fluorescent reporters were generated using genome editing, in order to investigate DNJ-14 expression across all cells in a living animal. C-terminal insertion of the fluorescent tag in the *dnj-14(wrmScarlet fusion)* strain did not interfere with DNJ-14 function, utilising the chemotaxis assay as a read-out of DNJ-14 function. This contrasts with the strong chemotaxis defect observed in the *dnj-14(wrmScarlet null)* strain created here and the previously described *dnj-14(null)* strain (Barker et al. 2023), both of which entirely lack DNJ-14 protein. Given that the chemotaxis deficits in these two independently generated *dnj-14* molecular null mutants correspond with those of earlier *dnj-14* ‘null’ mutants (Chen et al. 2015; Kashyap et al. 2014) and more recently developed autosomal dominant, adult-onset neuronal ceroid lipofuscinosis (ANCL) point mutants (Barker et al. 2023), we conclude that this is a robust and reproducible phenotype caused by impairment of DNJ-14 function. Furthermore, the observation that the DNJ-14-wrmScarlet fusion protein rescues chemotaxis to WT levels, whereas mistargeted ANCL point mutant DNJ-14 proteins fail to rescue this phenotype (Barker et al. 2023), strongly suggests that C-terminal tagging does not affect DNJ-14 membrane targeting. This is consistent with studies of mammalian CSPα demonstrating that C-terminal fusion with GFP or miniTurbo does not affect CSPα localisation (Barker et al. 2024; Sharma et al. 2011).

Background autofluorescence can be a problem for imaging in *C. elegans*, particularly in tissues such as the gut and in ageing studies across the life course. However, as wrmScarlet is the brightest currently available red fluorophore, this maximises signal-to-noise ratio and hence reduces the contribution of background autofluorescence. In addition, in our hands, gut autofluorescence at wavelengths used for wrmScarlet imaging was lower than those used for GFP. Finally, as red autofluorescence increases only gradually in ageing worms, whereas green and blue autofluorescence increases greatly preceding death (Pincus et al. 2016), wrmScarlet is advantageous for imaging over the life course. The expression of DNJ-14 was most obvious within the head/pharynx, intestine, spermatheca and the vulva/uterus upon initial observation. This has some similarity with the expression patterns of the gene adjacent to *dnj-14*, *glit-1*. Previous analysis of a *Pglit-1::GFP* transcriptional reporter and an N-terminal GFP-tagged *glit-1* translational reporter revealed expression in the pharynx, the intestine and several unidentified cells in the head (Offenburger et al. 2018). Given that *dnj-14* shares a promoter-encoding region with *glit-1* (Fig. 1), some similarity of expression is perhaps expected. However, the actual promoter elements may be different and the genes are transcribed from opposite strands. Indeed, their expression patterns are clearly not identical, with DNJ-14 also being observed in the vulva/uterus and spermathecae, unlike GLIT-1.

The pharyngeal expression of DNJ-14 was validated through co-localisation of fluorescence in the head with *Psod-3::GFP*, which is expressed in both the intestine and pharynx (MacNeil et al. 2015). It is unclear whether this reflects DNJ-14 expression in the pharyngeal muscles themselves, or in the surrounding pharyngeal nervous system. DNJ-14 fluorescence was also clearly present in various punctate structures outside the pharynx but within the head, which may represent the unidentified cells of the head previously observed with *glit-4* reporters. The intestinal-like pattern of DNJ-14 fluorescence was validated through co-localisation with GFP expressed under *sod-3* and *hsp-4* promoters, which serve as markers to the intestine and pharynx and to the intestine and hypodermis respectively (MacNeil et al. 2015). However, co-localisation with *sod-3* and *hsp-4* reporters was only partial, which could potentially be improved upon in future studies using additional intestinal markers such as *vha-6* or *cav-2*. Mammalian CSPα has been shown to localise to the digestive system previously, in its role in insulin secretion by β-cells of the pancreas (Brown et al. 1998), and is known to be expressed in pancreatic acinar cells, where it plays a role in regulating the secretion of digestive enzymes (Weng et al. 2009). Furthermore, CSPα is expressed in enterochromaffin-like cells of the gastric mucosa of the rat stomach (Zhao et al. 1997). Therefore, localisation to digestive tissues is not entirely surprising. However, the abundance of DNJ-14 expressed within the worm intestinal tract in comparison to the nervous system was somewhat unexpected.

The enrichment of DNJ-14 in worm reproductive tissues, namely the spermathecae and vulva/uterus, may also appear surprising, given the association of mammalian CSPα with neuronal tissues. However, in humans and mice, the less-studied CSPβ isoform is heavily expressed within the testis and plays a functional role in stabilising *trans*-SNARE complexes during sperm acrosomal exocytosis (Gorleku and Chamberlain 2010; Flores-Montero et al. 2022). The *C. elegans* spermatheca is the site of sperm storage and oocyte fertilisation (Lints and Hall 2009), but it is not clear if the observed DNJ-14 staining reflects expression in the spermathecal cells, or in the sperm stored within, or both. Nevertheless, since the worm genome contains only one CSP homologue, the expression of DNJ-14 reflects expression patterns in humans across the three CSP proteins. Hence, the high expression of CSPβ within the mammalian testis and of DNJ-14 in *C. elegans* spermathecae and vulva/uterus may suggest a conserved role for CSP proteins in reproductive tissues.

The expression of DNJ-14 observed here has some consistency with unbiased high-throughput mRNA expression analyses collated in WormBase (version 291, accessed January 2024). As expected, *dnj-14* gene expression is enriched in various neuronal cell types, including sensory neurons and GABAergic motor neurons, which may correspond to the DNJ-14::wrmScarlet staining in the head and ventral nerve cord, respectively. Interestingly, high *dnj-14* gene expression is also seen in the intestine, excretory cells and the rectal epithelium.

The extensive non-neuronal expression of DNJ-14 suggests that CSP proteins may have important physiological functions outside the nervous system that may have been overlooked due to the historical focus on neurons. This could potentially explain why only a small, partial rescue of the short lifespan of homozygous *csp*^*−/−*^* Drosophila* null mutants was observed when driving expression of either fly or human CSP with neuronal-specific promoters (Imler et al. 2019). These *Csp*^*−/−*^* Drosophila* rescued with neuronal CSP exhibited an average lifespan of only 15–20 days, at which point death of control heterozygous *Csp*^+*/−*^* Drosophila* was minimal. Given that *Csp* is expressed in various non-neuronal *Drosophila* tissues, this may suggest that the short lifespan phenotype of *csp* mutants is more influenced by non-neuronal than neuronal *Csp*. Similarly, in *C. elegans*, heterozygous *dnj-14* null mutants exhibited only a relatively mild chemotaxis defect, whereas they exhibited a large reduction in lifespan when compared with heterozygous ANCL mutants (Barker et al. 2023). It may be that this haploinsufficiency effect on lifespan reflects a requirement for non-neuronal CSP, which seems plausible given the well-established links between intestinal health and *C. elegans* lifespan (Hodge et al. 2022; Kumar et al. 2019). Taken together, these findings suggest there may be differential physiological roles for CSP in both neuronal and non-neuronal tissues, the latter of which has been largely ignored in the 30 years or so since CSP was discovered.

A general increase in DNJ-14 expression was observed across ageing, through an observed increase in wrmScarlet expression, which was particularly noticeable in the intestine. Indeed, proteomic profiling of primate synapses across normal healthy ageing reveals an increase in CSP expression in mid-age (40–50 years), compared with young age (18–25 years). This pattern is identified again when comparing those of mid-age (40–50 years) to old age (70 + years) (Graham et al. 2019). A much more striking increase in DNJ-14 expression was observed following food deprivation, which was not due to a generalised stress response. In starved animals, DNJ-14 expression was expressed more widely across the intestine and could be observed as large punctate structures surrounding the intestine. It is not entirely clear which cell types contain these punctae, but candidates include intestinal epithelial cells and coelomocytes. Intestinal fluorescence driven by the autophagy reporter GFP::LGG-1 forms similar punctate structures when starved, or when crossed with autophagy mutants (Zhang et al. 2015). Given that starvation is known to induce autophagy to promote survival in *C. elegans* (Kang et al. 2007), and the mirroring of expression with autophagic reporters following starvation, it is possible this increase in DNJ-14 expression in these punctate structures surrounding the intestine occurs as a result of starvation-induced autophagy. Indeed, mammalian CSPα has recently been implicated in microautophagy as a means of protein quality control (Hodge et al. 2022).

Since CSP’s initial discovery in the *Drosophila* brain and its known association with neurodegenerative disorders in humans, much of the work carried out on mammalian CSPα has been in a neuronal context. However, the high expression of DNJ-14 within the digestive system in *C. elegans*, and of CSPα in the oesophagus and pancreas in humans, suggests that CSPα may play an important role in the digestive system. However, the understanding of the role of CSPα in the context of the digestive tract is currently limited. Therefore, future studies investigating the role of CSPα in non-neuronal mammalian tissues would be beneficial in gaining a well-rounded overview into CSPα function. Furthermore, the possibility that some ANCL phenotypes may occur as either a direct or indirect result of alterations in function of non-neuronal human CSPα warrants further attention.

### Supplementary Information

Below is the link to the electronic supplementary material.

Supplementary file1 (PDF 932 KB)

## Data Availability

The authors confirm that the data supporting the findings of this study are available within the article and its supplementary materials. All C. elegans strains created in this study are freely available upon request.
