# Supplementary material for: Tissue distribution of cysteine string protein/DNAJC5 in C. elegans analysed by CRISPR/Cas9-mediated tagging of endogenous DNJ-14
Source: Cell Tissue Res. 2024 Feb 26;396(1):41–55. doi: 10.1007/s00441-024-03875-w (PMC10997724; doi:10.1007/s00441-024-03875-w)
Supplement: Supplementary file 1 — Supplementary file1 (PDF 932 KB) [file 441_2024_3875_MOESM1_ESM.pdf]

Supplementary Table 1 Microscope configurations and image-acquisition parameters

**Microscope component**	**Parameters to report**
**Microscope**	Leica DMi8 with Andor Dragonfly
**Light source**	7-line integrated laser engine equipped with:<br>Solid state 488 smart diode laser at 50mW: set to 2.0 %<br>OBIS LS 561 smart OPSS laser at 50mW: set to 5.0 %
**Excitation/emission optics**	Dichroic mirror: Quad EM filter 405-488-561-640<br>Dual camera beam splitter: Dual camera dichroic 565nm long pass<br>Emission filters: 525/50nm bandpass filter<br>600/50nm bandpass filter<br>spinning disk with 40 µm pinholes
**Objective lenses**	Leica objectives:<br>11506358 HC PL APO 40X/1.30 OIL CS2<br>11506319 HC PL APO 63X/1.47 OIL CORR TIRF
**Detector**	Andor iXon Ultra 888 Ultra EMCCD Camera<br>1024 × 1024;<br>488: 100 ms exposure; EM gain 300<br>561: 100 ms exposure; EM gain 300<br>Averaging: 1; Binning: 1; camera magnification 1X (40X objective) or 2X (63X objective)<br>Nyquist Z sampling

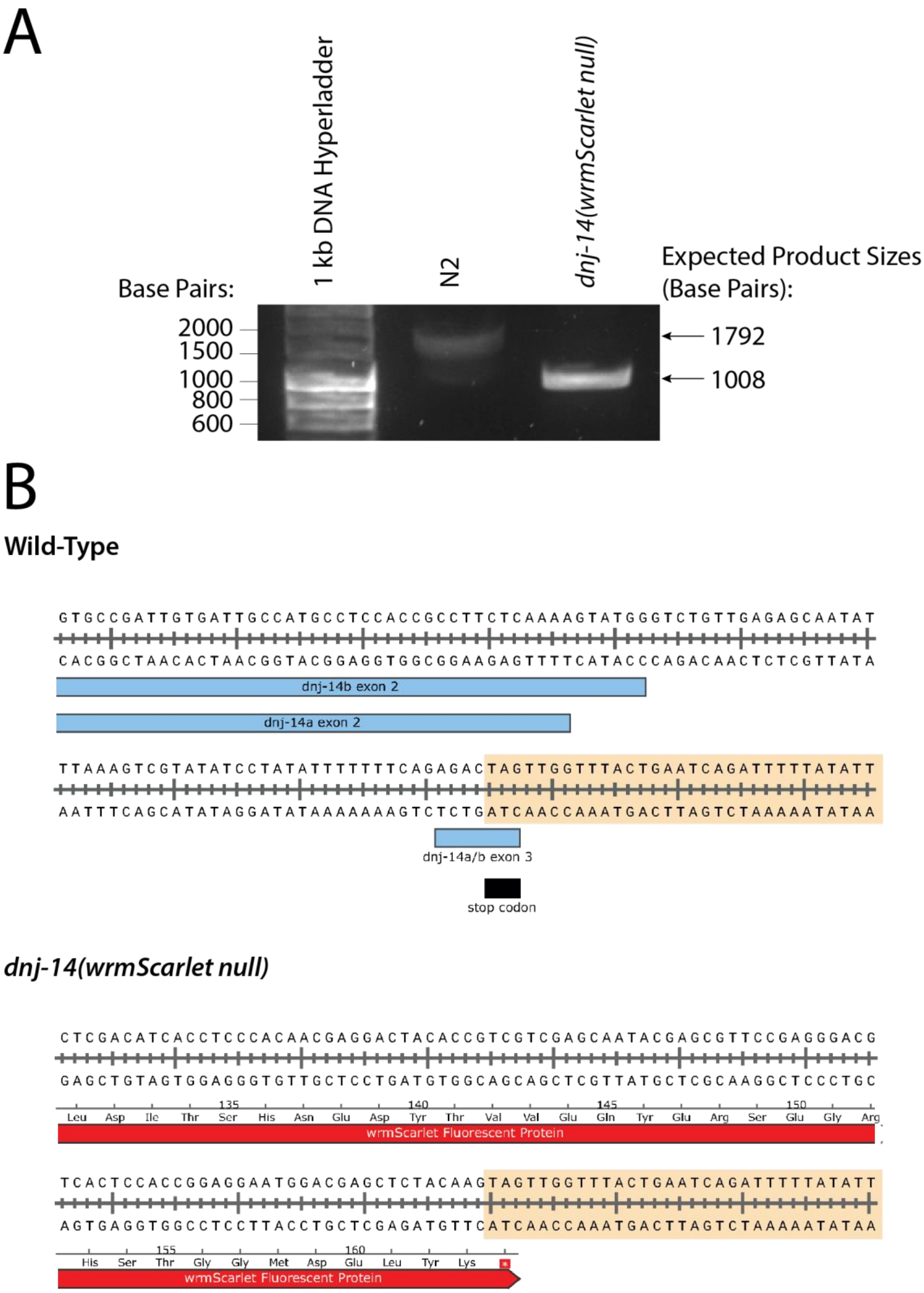

Supplementary Figure S1 **Genotyping results of *dnj-14(wrmScarlet null) C. elegans*.**
(A) Genotyping PCR performed on N2 WT and *dnj-14(wrmScarlet null) C. elegans.* Expected product size for the *dnj-14(wrmScarlet null)* is 784 bp smaller than that of N2 worms, due to the loss of a 1477 bp sequence encoding *dnj-14,* and addition of a 693 bp sequence encoding the wrmScarlet protein. Sequencing results of *dnj-14*(*wrmScarlet null),* showing successful integration of wrmScarlet fluorescent protein in place of *dnj-14*, in comparison to WT N2 sequence. Complimentary sequences are highlighted to show their relative positions in the genome.

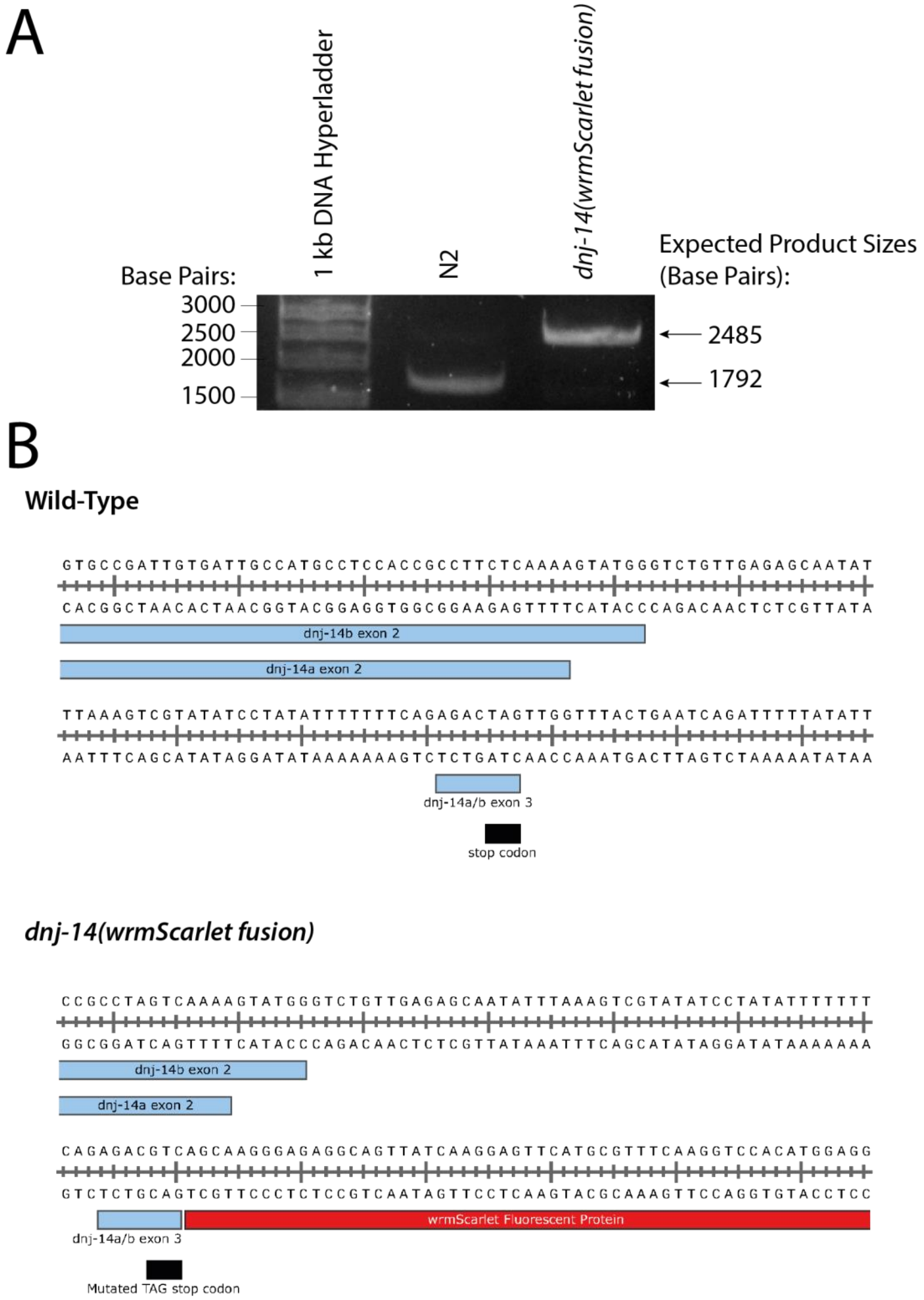

Supplementary Figure S2 Genotyping results of *dnj-14(wrmScarlet fusion) C. elegans*.
(A) Genotyping PCR performed on N2 WT and *dnj-14(wrmScarlet fusion) C. elegans.* Expected product size for the *dnj-14*(*wrmScarlet fusion)* is 693 bp larger than that of N2 WT worms, due to the addition of a 693 bp sequence encoding the wrmScarlet protein. (B) Sequencing results of *dnj-14*(*wrmScarlet fusion),* showing successful integration of wrmScarlet fluorescent protein at the C-terminus of *dnj-14.*

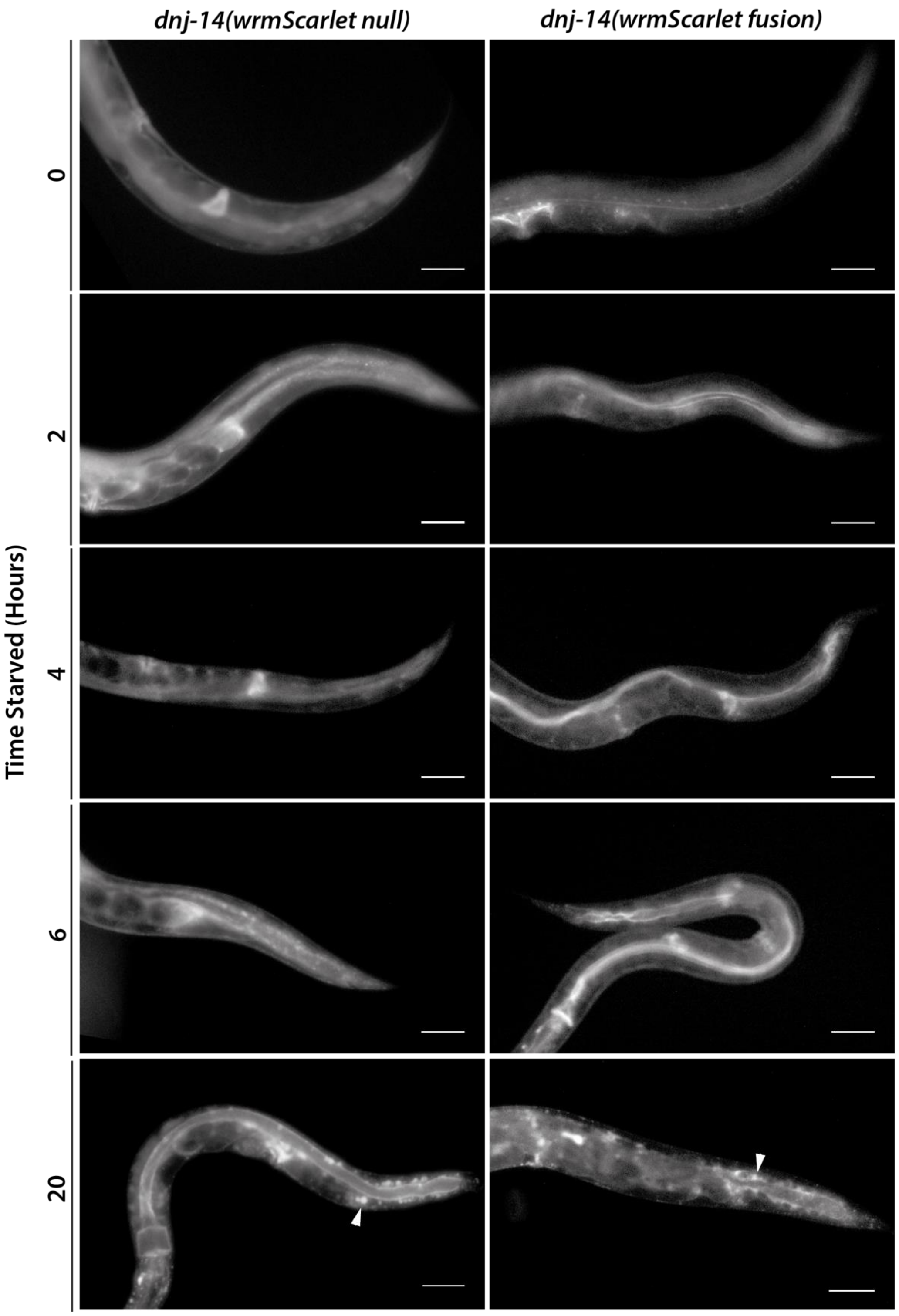

Supplementary Figure S3 Changes in DNJ-14 expression occur following 20 hours of food deprivation. Compared here are representative images of *dnj-14(wrmScarlet null)* and *dnj-14(wrmScarlet fusion) C. elegans*. Worms were completely removed from a OP50 bacterial food source for 0–20 hours. Images were acquired on a Nikon Eclipse-Ti inverted fluorescence microscope, with NIS-Elements microscope imaging software, using 20× objective lens. Note that at 20 hours starvation, there are changes in wrmScarlet expression (denoted by arrowheads), which are not apparent in any of the other corresponding images (0—6 hours starved). Scale bars: 50 μm.

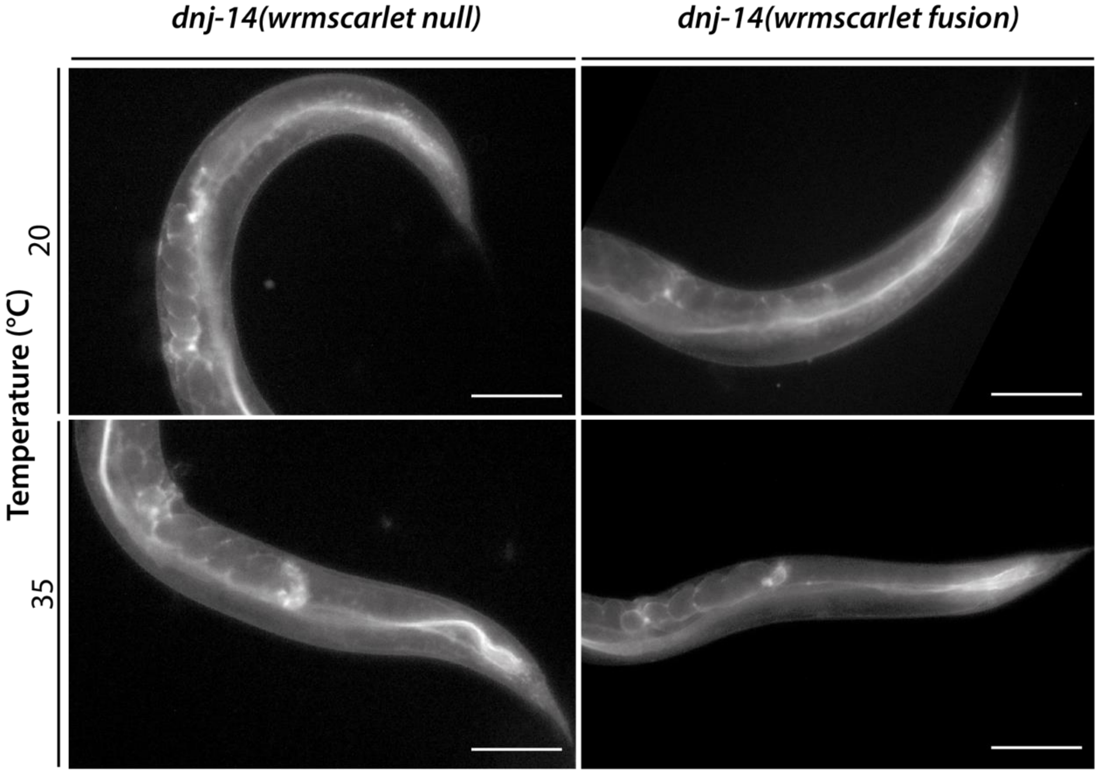

Supplementary Figure S4 DNJ-14 expression does not change following heat shock. Age-synchronised adult day 1 *dnj-14(wrmScarlet null)* and *dnj-14(wrmScarlet fusion) C. elegans* were subject to heat shocking at 35°C for 2 hours, 24 hours prior to imaging, using worms kept at 20°C as a control. Images were acquired on a Nikon Eclipse-Ti inverted fluorescence microscope, with NIS-Elements microscope imaging software, using a 20× objective lens. Scale bars: 100 μm.

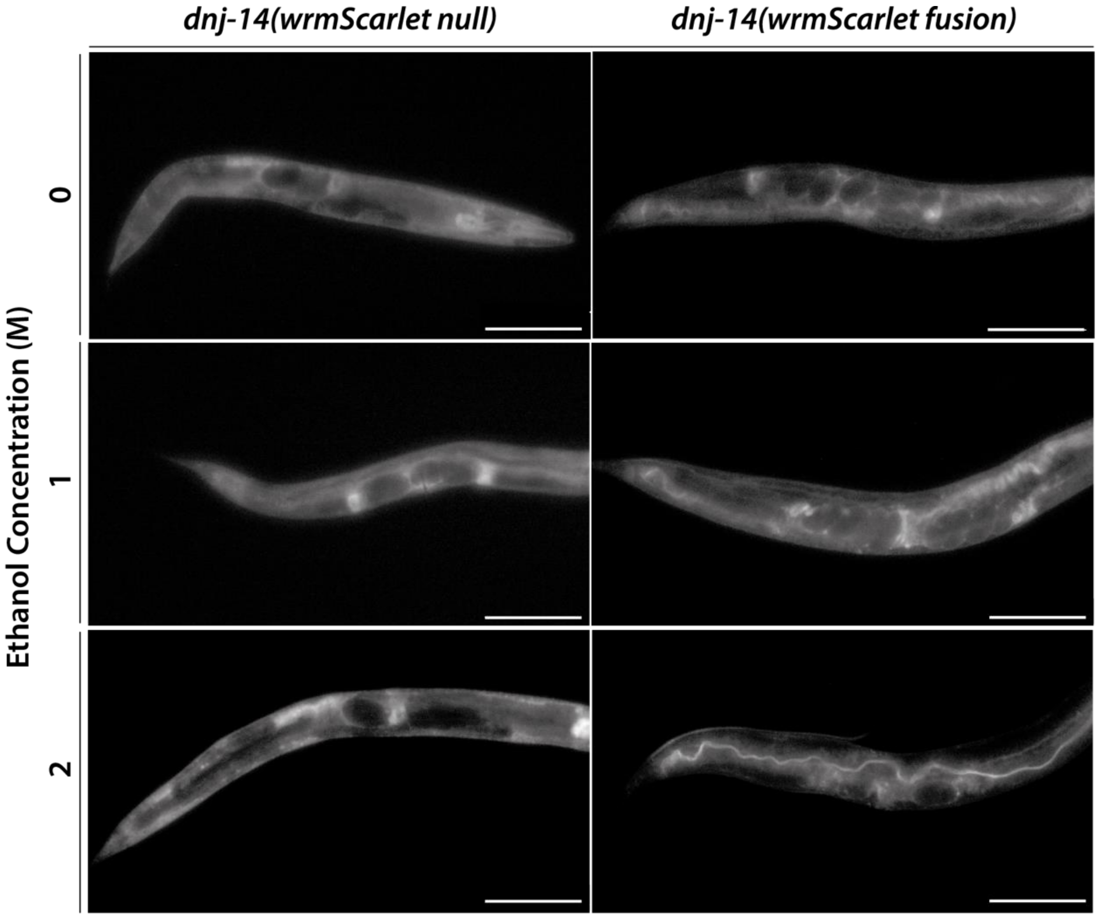

Supplementary Figure S5 DNJ-14 expression does not change following osmotic shock. Young adult *dnj-14(wrmScarlet null)* and *dnj-14(wrmScarlet fusion) C. elegans* were were incubated with 0 (control), 1 M or 2 M ethanol for 2 hours, 24 hours prior to imaging. Images were acquired on a Nikon Eclipse-Ti inverted fluorescence microscope, with NIS-Elements microscope imaging software, using a 20× objective lens. Scale bars: 100 μm.
